# Correlates of 25-Hydroxyvitamin D among Chinese Breast Cancer Patients

**DOI:** 10.1371/journal.pone.0086467

**Published:** 2014-01-21

**Authors:** Liang Shi, Sarah Nechuta, Yu-Tang Gao, Ying Zheng, Tsogzolmaa Dorjgochoo, Jie Wu, Qiuyin Cai, Wei Zheng, Wei Lu, Xiao Ou Shu

**Affiliations:** 1 Department of Medicine, Division of Epidemiology, Vanderbilt University School of Medicine, Nashville, Tennessee, United States of America; 2 Department of Diabetes Control and Prevention, Shanghai Municipal Center for Disease Control and Prevention, Shanghai, China; 3 Department of Cancer Prevention & Control, Shanghai Municipal Center for Disease Control and Prevention, Shanghai, China; 4 Department of Epidemiology, Shanghai Cancer Institute, Shanghai, China; MOE Key Laboratory of Environment and Health, School of Public Health, Tongji Medical College, Huazhong University of Science and Technology, China

## Abstract

**Background:**

Few studies have investigated vitamin D status in association with modifiable lifestyle factors and clinical characteristics among breast cancer patients, with no studies among Chinese women, who may be at higher risk of vitamin D deficiency. We aimed to evaluate circulating 25-hydroxyvitamin D (25(OH)D) levels in association with clinical and lifestyle factors among 1,940 Chinese breast cancer patients.

**Methods:**

Participants included breast cancer cases aged 22–77 from a population-based case-control study conducted in Shanghai, China during 1996–1998 (n = 1,044) and 2002–2005 (n = 896). Circulating 25(OH)D levels were measured in plasma samples (95% collected ≤6 months post-diagnosis). Prevalence ORs and 95% CIs were derived from multinomial logistic regression models, adjusting for age, season, and other factors.

**Results:**

About 23% and 48% of women were vitamin D deficient (<30 nmol/L) or insufficient (30–50 nmol/L), respectively. Tumor characteristics were not associated with vitamin D status. Higher BMI was associated with increased odds of vitamin D deficiency (ORs (95% CIs): 1 (reference), 1.12 (0.85,1.47), and 1.57 (1.02,2.42), for <23, 23–<27.5, and ≥27.5 kg/m^2^, respectively, P_trend_ <0.06). Total physical activity was associated with reduced odds of vitamin D deficiency (ORs (95% CIs):1 (reference), 0.84 (0.59,1.20), 0.65 (0.45,0.93), and 0.69 (0.48,1.00), for <7.65, 7.65–<10.6, 10.6–<13.5, ≥13.5 MET-hours/day, respectively, P_trend_ <0.02). Smoking was associated with vitamin D insufficiency and deficiency (ORs (95% CIs): 2.50 (1.07,5.84) and 2.78 (1.11,6.95), respectively).

**Conclusions:**

In the largest study to date, the prevalence of low vitamin D status was high among Chinese breast cancer patients and associated with higher BMI, smoking, and lower physical activity. Our findings support careful monitoring of vitamin D status and recommendations for supplementation and other lifestyle modifications that may improve vitamin D status in breast cancer patients.

## Introduction

Vitamin D is an essential nutrient, and plays an important role in calcium-phosphorus homeostasis, bone metabolism, immune function, and cellular growth, differentiation and apoptosis [1], [2]. Vitamin D is obtained from exposure to sunlight and through dietary sources including food and supplements. In recent years, epidemiologic studies have linked vitamin D deficiency with a number of adverse outcomes, including cardiovascular and cancer-related morbidity and mortality [3]–[7].

Low circulating vitamin D levels may be associated with advanced breast cancer stage, and breast cancer recurrence and mortality [8]–[12]. Vitamin D plays a key role in bone health, and breast cancer survivors are at increased risk for bone loss and fractures, potentially due to cancer treatment long-term effects [5], [6]. In addition, low vitamin D status is associated with obesity, and weight gain is common among breast cancer survivors. Further, vitamin D deficiency is often unrecognized by clinicians [13]. Therefore, low vitamin D status is a particular concern among breast cancer survivors. However, few large studies to date have investigated correlates of vitamin D status, including modifiable lifestyle-related factors (e.g., obesity, physical activity) and clinical characteristics, among breast cancer patients, and studies were limited to Western populations [8], [12], [14]–[16]. Using data on 1,940 breast cancer patients originally recruited to participate in a large population-based case-control study conducted in Shanghai, China, we measured post-diagnosis circulating levels of 25-hydroxyvitamin D (25(OH)D) to evaluate vitamin D status, and associations between vitamin D status and lifestyle factors and clinical characteristics.

## Materials and Methods

### Ethics Statement

The study was approved by the institutional review boards of Vanderbilt University, Nashville, TN and the Shanghai Cancer Institute, Shanghai, China. Before interviews were conducted, written informed consent was obtained from all patients.

### Study Population

Participants of the present study included breast cancer cases recruited to the Shanghai Breast Cancer Study (SBCS), a two-phase population-based case-control study conducted in Shanghai, China. A detailed description of the case-control study methodology has been previously published [17]. Briefly, 3,454 incident breast cancer patients and 3,474 community controls were recruited into the SBCS between 1996 and 1998 (Phase I; 1,459 cases; response rate: 90.3%) and between 2002 and 2005 (Phase II; 1,989 cases; response rate: 83.7%). Cases who donated a blood sample and were followed for breast cancer outcomes were included in the present study (1,045 from Phase I (71.6%) and 897 from Phase II (45%)), as described previously [18].

### Data Collection

Information collected using structured questionnaires during in-person interviews included socio-demographics, medical history, family history of cancer, dietary habits, lifestyle habits (e.g., physical activity, smoking, alcohol intake), and reproductive history. Medical charts were reviewed to obtain treatment information including data on cancer diagnosis, tumor-node-metastasis (TNM) disease stage, and estrogen receptor and progesterone receptor status. Weight, waist circumference, hip circumference, and height were measured by trained interviewers according to a standard protocol.

Women were queried regarding their regular physical activity habits for the previous ten years before their breast cancer diagnosis. Specifically, women reported the average duration (minutes/day) and the length of participation (years) for up to five exercise or sport activities. Total energy expenditure was estimated by the summation of energy expended in each activity reported using standardized metabolic equivalent values (METs)-hours/day/year [19]. Participants were also asked to report the duration (minutes) they spent walking and cycling for transportation each day, and the duration (hours/week) they spent doing housework. Energy expenditure for non-exercise activities was estimated using the following standard METs: housework (2.0 METs); walking (3.3 METs) and bicycling (4.0 METs) [19], [20]. Total physical activity MET-hours/day was calculated by combining exercise/sport and non-exercise MET-hours/day.

### Vitamin D Measurement and Definition

Circulating 25(OH)D concentrations were measured in stored plasma (−80°C) via chemiluminescent Immunoassay (Heartland Assays, Inc. Ames, Iowa) [21]. Blinded quality control pooled samples (n = 45) and vitamin D standard reference samples (level 1) from the National Institute for Standards and Technology [22] (n = 20) were included. The inter-assay coefficient of variation for 25(OH)D measurements was 7.72% in our study samples and 6.56% in the National Institute for Standards and Technology samples.

Various definitions of vitamin D insufficiency and deficiency have been suggested. We used the most recent cutpoints recommended by Institute of Medicine (2011) [23], with vitamin D deficiency defined as 25(OH)D concentrations <30 nmol/L, vitamin D insufficiency defined as 25(OH)D concentrations between 30–50 nmol/L, and vitamin D sufficiency defined as 25(OH)D concentrations >50 nmol/L.

### Statistical Analysis

Two women missing vitamin D measurements were excluded for a final analytic sample of 1,940 participants. Differences in socio-demographic and other characteristics of breast cancer patients by vitamin D status were evaluated using the Kruskal-Wallis test for continuous variables and Chi-square test for categorical variables. Factors evaluated included: age at diagnosis, education, family income in Yuan, occupation, season of blood draw, time between diagnosis and blood draw, data source (i.e., SBCS phase I or phase II), menopausal status, TNM stage, estrogen receptor/progesterone receptor status, waist circumference, waist-to-hip ratio (WHR), body mass index (BMI), total physical activity in MET-hours/day, regular exercise (minutes/day), walking for transportation (minutes/day), biking for transportation (minutes/day), ever regularly smoking (>1 cigarette per day for >6 months), ever regularly drinking alcohol (>1 drink per week for >6 months). Multivariable multinomial logistic regression models were used to calculate (1) age- and season-adjusted and (2) further-adjusted prevalence odd ratios (ORs) and 95% confidence intervals (CIs) for associations of clinical characteristics and lifestyle-related factors with vitamin D status (deficient, insufficient, sufficient (reference group)). In multivariable analyses, waist circumference, WHR, BMI, and total physical activity were categorized based on quartiles. BMI was also categorized based on WHO and Asian-specific cutpoints [24]. We also created a variable using duration of exercise and transportation physical activity (walking and biking) as an estimate of outdoor activity. Factors previously noted to be associated with vitamin D status or adjusted for in previous reports were controlled in further adjusted models, including education, SBCS study phase, BMI, total physical activity, menopausal status, TNM stage, and ER/PR status. As 25(OH)D levels were measured prior to initiation of radiotherapy and chemotherapy for most participants, we did not investigate associations between vitamin D status and breast cancer treatment. As a secondary aim, for a subset of participants with available data (n = 896, phase II cases), we investigated the association between two major comorbidities (hypertension and diabetes) and regular use of supplements that may contain vitamin D (calcium and multivitamins) in the past five years (regular indicated use ≥three times per week for >two months continuously). All statistical tests were two-sided and considered to be statistically significant if the P-value was <0.05. All statistical analyses were performed using SAS software 9.3 (SAS Institute, Cary, NC, USA).

## Results

The mean age at diagnosis of our study participants (n = 1,940) was 49.6 years (range: 22–77 years). Approximately 95% of blood samples were collected during the first 6 months after breast cancer diagnosis (74.3% within 1 month). The mean 25(OH)D concentration was 42.5 nmol/L (SD = 16.2). Approximately 23% of patients were classified as vitamin D deficient and 48% were classified as vitamin D insufficient. Only 28% of patients were classified as vitamin D sufficient. Data on receipt (yes, no) of radiotherapy, chemotherapy, and tamoxifen therapy were available for 1,784 (92.0%), 1,920 (99.0%) and 1,646 (84.8%) of participants, respectively. Excluding women who were missing data, 93.7% received chemotherapy, 36.2% received radiotherapy, and 67.1% received tamoxifen.

**Table 1** displays selected cohort characteristics by vitamin D status. Patients with higher concentrations of vitamin D tended to be younger, have their vitamin D levels measured in the summer or autumn, and be premenopausal. Measures of socioeconomic status (i.e., education, income, and occupation), stage, tumor hormone receptor status, and time between diagnosis and blood draw were not significantly associated with vitamin D status.

**Table 1:** Select Cohort Characteristics by Vitamin D Status (n = 1,940).

	25(OH)D levels (nmol/L)			
	Deficient (<30) N = 450	Insufficient (30–50) N = 938	Sufficient (>50) N = 552	P value^a^
**Age at interview,** mean (±SD)	50.3± (8.1)	50.2± (8.5)	48.8± (7.9)	<0.01
**Education**, n (%)				
None/elementary	45 (10.0)	97 (10.3)	47 (8.5)	
Middle school	193 (42.9)	426 (45.4)	239 (43.3)	
High school	159 (35.3)	307 (32.7)	198 (35.9)	
≥College	53 (11.8)	108 (11.5)	68 (12.3)	0.49
**Family Income (Yuan)**, n (%)				
<10000	140 (31.1)	294 (31.3)	188 (34.1)	
10000–19999	159 (35.3)	380 (40.5)	195 (35.3)	
20000–29999	80 (17.8)	128 (13.7)	92 (16.7)	
≥30000	71 (15.8)	136 (14.5)	77 (14.0)	0.25
**Occupation**, n (%)^b^				
Professional	119 (26.5)	225 (24.0)	138 (25.1)	
Clerical	107 (23.8)	223 (23.8)	144 (26.1)	
Manual worker/housewife	223 (49.7)	489 (52.2)	269 (48.8)	0.98
**Season of blood draw**, n (%)				
Winter	120 (26.7)	207 (22.1)	112 (20.3)	
Spring	133 (29.6)	266 (28.4)	132 (23.9)	
Summer	107 (23.8)	238 (25.4)	149 (27.0)	
Autumn	90 (20.0)	227 (24.2)	159 (28.8)	<0.01
**Time between diagnosis and blood draw** (months), n (%)				
≤1	346 (76.9)	690 (73.6)	406 (73.6)	
1–<6	86 (19.1)	196 (20.9)	117 (21.2)	
≥6	18 (4.0)	52 (5.5)	29 (5.2)	0.22
**Data source**, n (%)				
SBCS I	257 (57.1)	488 (52.0)	299 (54.2)	
SBCS II	193 (42.9)	450 (48.0)	253 (45.8)	0.20
**Menopausal status**, n (%)				
Premenopausal	265 (58.9)	557 (59.4)	366 (66.3)	
Postmenopausal	185 (41.1)	381 (40.6)	186 (33.7)	0.02
**TNM stage**, n (%)				
0–I	128 (28.4)	279 (29.7)	158 (28.6)	
II	231 (51.3)	496 (52.9)	282 (51.1)	
III–IV	49 (10.9)	94 (10)	43 (7.8)	
Unknown	42 (9.3)	69 (7.4)	69 (12.5)	0.49
**Hormone receptor status**, n (%)				
ER+/PR+	198 (44.0)	404 (43.1)	248 (44.9)	
ER+/PR- or ER−/PR+	74 (16.4)	152 (16.2)	91 (16.5)	
ER−/PR-	85 (18.9)	215 (22.9)	111 (20.1)	
Unknown	93 (20.7)	167 (17.8)	102 (18.5)	0.57

a Based on Chi-square test for categorical variables and Kruskal-Wallis Test for continuous variables.

b Excludes three women missing occupation.

**Table 2** displays prevalence ORs (95% CIs) for associations of anthropometric factors and vitamin D status. Compared to the lowest BMI category, the highest BMI category based on quartiles (≥25.9 kg/m^2^) or Asian-specific cutpoints (≥27.5 kg/m^2^) was associated with vitamin D deficiency in both (1) age- and season-adjusted models and (2) models additionally adjusted for education, SBCS phase, total physical activity, menopausal status, TMN stage, and hormone receptor status. For example, a BMI ≥27.5 kg/m^2^ (vs. <23 kg/m^2^) was associated with a 57% increased odds for vitamin D deficiency (fully-adjusted OR = 1.57, 95% CI: 1.02, 2.42, P_trend_ = 0.06). BMI was not significantly associated with vitamin D insufficiency status. Compared to a waist circumference of <73.0 cm, a waist circumference of 73–<79 cm was inversely associated with vitamin D deficiency in both age- and season-adjusted and further-adjusted models. However, higher waist circumference (>79 cm) and WHR were not associated with vitamin D status. Associations for the central adiposity measures with vitamin D status were similar when BMI was included in the models (data not shown). Tumor characteristics and menopausal status were not associated with vitamin D status in age- and season-adjusted or further-adjusted models (data not shown).

**Table 2:** Prevalence ORs and 95% CIs for associations of anthropometric factors with vitamin D status^a^ among breast cancer patients (n = 1,940).

		Adjusted for age and season						Further adjusted^c^			
	Sufficient^b^ N = 552	Insufficient (30–50) N = 938			Deficient (<30) N = 450			Insufficient (30–50) N = 938		Deficient (<30) N = 450	
	NO.	NO.	OR	(95% CI)	NO.	OR	(95% CI)	OR	(95% CI)	OR	(95% CI)
**Waist (cm)**											
<73.0	125	209	1.00	(reference)	103	1.00	(reference)	1.00	(reference)	1.00	(reference)
73.0–<79.0	167	245	0.83	(0.61,1.12)	97	0.66	(0.46,0.96)	0.81	(0.60,1.10)	0.66	(0.46,0.96)
79.0–<85.0	132	232	0.96	(0.70,1.31)	112	0.94	(0.65,1.37)	0.94	(0.68,1.29)	0.96	(0.66,1.40)
≥85.0	128	252	1.02	(0.74,1.41)	137	1.14	(0.78,1.67)	0.98	(0.70,1.37)	1.18	(0.80,1.74)
P_trend_			0.66			0.16		0.83		0.12	
**WHR**											
<0.78	106	194	1.00	(reference)	87	1.00	(reference)	1.00	(reference)	1.00	(reference)
0.78–<0.82	160	237	0.78	(0.57,1.07)	98	0.73	(0.50,1.08)	0.77	(0.56,1.05)	0.77	(0.52,1.13)
0.82–<0.86	150	230	0.78	(0.56,1.07)	116	0.90	(0.61,1.32)	0.76	(0.55,1.05)	0.94	(0.64,1.39)
≥0.86	136	275	0.96	(0.69,1.33)	148	1.18	(0.80,1.74)	0.91	(0.65,1.28)	1.27	(0.85,1.91)
P_trend_			0.95			0.16		0.71		0.09	
**BMI Quartiles (kg/m^2^)**											
<21.5	143	229	1.00	(reference)	99	1.00	(reference)	1.00	(reference)	1.00	(reference)
21.5–<23.5	146	225	0.93	(0.69,1.25)	114	1.09	(0.76,1.57)	0.91	(0.68,1.23)	1.10	(0.77,1.59)
23.5–<25.9	147	249	0.99	(0.74,1.33)	107	0.99	(0.69,1.42)	0.97	(0.72,1.32)	1.00	(0.69,1.44)
≥25.9	116	234	1.14	(0.83,1.57)	129	1.47	(1.01,2.13)	1.13	(0.82,1.56)	1.50	(1.03,2.20)
P_trend_			0.39			0.08		0.43		0.07	
**BMI WHO categories (kg/m^2^)**											
<25	384	633	1.00	(reference)	282	1.00	(reference)	1.00	(reference)	1.00	(reference)
25–<30	147	257	1.01	(0.79,1.29)	143	1.28	(0.96,1.69)	1.00	(0.78,1.28)	1.29	(0.97,1.72)
≥30	21	47	1.21	(0.71,2.07)	24	1.40	(0.76,2.59)	1.21	(0.70,2.08)	1.41	(0.76,2.63)
P_trend_			0.62			0.07		0.66		0.07	
**BMI Asian categories (kg/m^2^)**											
<23	253	388	1.00	(reference)	181	1.00	(reference)	1.00	(reference)	1.00	(reference)
23–<27.5	245	425	1.08	(0.86,1.36)	203	1.11	(0.85,1.46)	1.07	(0.85,1.35)	1.12	(0.85,1.47)
≥27.5	54	124	1.35	(0.94,1.95)	65	1.53	(1.01,2.34)	1.35	(0.93,1.97)	1.57	(1.02,2.42)
P_trend_			0.13			0.07		0.15		0.06	

a Vitamin D status was based on measured circulating 25(OH)D levels in nmol/L. Women missing anthropometric data were excluded: waist (n = 1), WHR (n = 3), BMI (n = 2).

b Reference group.

c Additionally adjusted for (where applicable) education level, SBCS phase, total physical activity (MET-hours/day), menopausal status, TNM stage, tumor estrogen/progesterone receptor status.

**Table 3** displays prevalence ORs (95% CIs) for associations of physical activity, smoking, and alcohol drinking with vitamin D status. In age-and season-adjusted models, higher total physical activity in MET-hours/day was associated with reduced odds of vitamin D deficiency (P_trend_ = 0.02), but was not associated with vitamin D insufficiency (P_trend_ = 0.67). Results were similar in further-adjusted models. Regular exercise participation was not significantly associated with vitamin D status, however samples sizes were small as only 24.5% of breast cancer patients reported regular exercise prior to diagnosis (and only 7.2% reported ≥30 minutes/day of regular exercise). Walking for transportation was not significantly associated with vitamin D status, while biking for transportation was associated with a decreased odds for vitamin D deficiency (further-adjusted OR for ≥60 minutes/day (vs. none) = 0.56, 95% CI: 0.38, 0.82, P_trend_ <0.01). Biking for transportation was also associated with vitamin D insufficiency, however, only for women who biked <30 minutes/day vs. none (further-adjusted OR = 0.53, 95% CI: 0.33, 0.87, P_trend_ = 0.44). Smoking and alcohol drinking rates are low among women in Shanghai, and few breast cancer patients reported regularly smoking (2.7%) or drinking alcohol (4.3%). Ever smoking was associated with vitamin D insufficiency and deficiency (further-adjusted ORs (95% CIs): 2.50 (1.07, 5.84) and 2.78 (1.11, 6.95), respectively). Alcohol consumption was not significantly associated with vitamin D status. We also conducted a multiple linear regression analysis with 25(OH)D as a continuous variable, and found similar associations for anthropometric factors, physical activity, smoking, and alcohol (**Table S1**).

**Table 3:** Prevalence ORs and 95% CIs for associations of physical activity, smoking, and alcohol drinking with vitamin D status^a^ among breast cancer patients (n = 1,940).

		Adjusted for age and season						Further adjusted^c^			
	Sufficient^b^ N = 552	Insufficient (30–50) N = 938			Deficient (<30) N = 450			Insufficient (30–50) N = 938		Deficient (<30) N = 450	
	NO.	NO.	OR	(95% CI)	NO.	OR	(95% CI)	OR	(95% CI)	OR	(95% CI)
**Total Physical Activity (MET-hours/day)**											
<7.65	135	221	1.00	(reference)	129	1.00	(reference)	1.00	(reference)	1.00	(reference)
7.65–<10.6	131	236	1.05	(0.78,1.43)	116	0.88	(0.62,1.25)	1.03	(0.76,1.40)	0.84	(0.59,1.20)
10.6–<13.5	149	236	0.91	(0.67,1.23)	101	0.66	(0.46,0.94)	0.87	(0.64,1.18)	0.65	(0.45,0.93)
≥13.5	137	245	0.98	(0.72,1.33)	104	0.69	(0.48,1.00)	0.94	(0.69,1.29)	0.69	(0.48,1.00)
P_trend_			0.67			0.02		0.48		0.02	
**Exercise/sports duration (minutes/day)** d											
none	426	695	1.00	(reference)	344	1.00	(reference)	1.00	(reference)	1.00	(reference)
<30	84	168	1.10	(0.82,1.48)	79	1.00	(0.70,1.41)	1.10	(0.82,1.49)	1.00	(0.70,1.43)
≥30	39	74	0.96	(0.63,1.47)	26	0.64	(0.38,1.10)	0.96	(0.62,1.48)	0.69	(0.40,1.20)
P_trend_			0.89			0.20		0.86		0.31	
**Walking for transportation (minutes/day)** e,f											
<30	120	162	1.00	(reference)	80	1.00	(reference)	1.00	(reference)	1.00	(reference)
30–59	141	253	1.25	(0.91,1.71)	122	1.20	(0.82,1.75)	1.18	(0.85,1.65)	1.04	(0.70,1.54)
59–90	177	277	1.05	(0.77,1.44)	145	1.11	(0.76,1.60)	0.98	(0.70,1.37)	0.91	(0.61,1.35)
≥90	114	246	1.43	(1.03,2.00)	103	1.20	(0.80,1.79)	1.32	(0.92,1.89)	1.00	(0.65,1.54)
P_trend_			0.11			0.54		0.30		0.81	
**Biking for transportation (minutes/day)** f,g											
none	334	626	1.00	(reference)	341	1.00	(reference)	1.00	(reference)	1.00	(reference)
<30	41	36	0.52	(0.33,0.84)	14	0.37	(0.20,0.70)	0.53	(0.33,0.87)	0.39	(0.20,0.73)
30–59	73	133	1.05	(0.76,1.45)	41	0.58	(0.38,0.88)	1.06	(0.76,1.48)	0.59	(0.39,0.91)
≥60	104	143	0.80	(0.60,1.08)	54	0.55	(0.38,0.80)	0.84	(0.62,1.14)	0.56	(0.38,0.82)
P_trend_			0.26			<0.01		0.44		<0.01	
**Transportation duration (minutes/day)** h,i											
<30	44	68	1.00	(reference)	48	1.00	(reference)	1.00	(reference)	1.00	(reference)
30–59	137	211	0.96	(0.62,1.49)	106	0.69	(0.42,1.12)	0.94	(0.60,1.46)	0.68	(0.41,1.11)
60–90	175	288	1.00	(0.65,1.53)	150	0.74	(0.46,1.18)	0.97	(0.63,1.50)	0.74	(0.46,1.20)
≥90	196	371	1.16	(0.76,1.76)	146	0.65	(0.40,1.03)	1.12	(0.73,1.73)	0.66	(0.41,1.07)
P_trend_			0.22			0.16		0.29		0.26	
**Estimated outdoor physical activity (minutes/day)** j											
<30	42	63	1.00	(reference)	47	1.00	(reference)	1.00	(reference)	1.00	(reference)
30–59	127	202	1.03	(0.66,1.62)	100	0.68	(0.41,1.12)	1.00	(0.63,1.58)	0.67	(0.40,1.10)
60–119	252	404	1.00	(0.65,1.53)	202	0.66	(0.42,1.05)	0.97	(0.63,1.50)	0.67	(0.42,1.07)
≥120	131	269	1.25	(0.80,1.96)	101	0.62	(0.38,1.02)	1.22	(0.77,1.93)	0.64	(0.38,1.07)
P_trend_			0.22			0.12		0.27		0.20	
**Ever regularly smoking**											
Never	545	909	1.00	(reference)	434	1.00	(reference)	1.00	(reference)	1.00	(reference)
Yes	7	29	2.40	(1.04,5.54)	16	2.72	(1.10,6.71)	2.50	(1.07,5.84)	2.78	(1.11,6.95)
**Ever regularly drinking**											
Never	534	895	1.00	(reference)	427	1.00	(reference)	1.00	(reference)	1.00	(reference)
Yes	18	43	1.45	(0.82,2.54)	23	1.62	(0.86,3.05)	1.51	(0.85,2.66)	1.70	(0.89,3.22)

a Vitamin D status was based on measured circulating 25(OH)D levels in nmol/L. Models with exercise duration exclude five women missing data on exercise duration.

b Reference group.

c Additionally adjusted for education level, SBCS phase, BMI in quartiles, menopausal status, TNM stage, tumor estrogen/progesterone receptor status and total physical activity (MET-hours/day) (where applicable).

d Additionally adjusted for non-exercise METs.

e Did not include walking for exercise.

f Additionally adjusted for exercise duration, walking duration (where applicable), and biking duration (where applicable).

g Did not include biking for exercise.

h Walking (not for exercise) and biking (not for exercise) duration.

i Additionally adjusted for exercise duration.

j Includes walking and biking for transportation and exercise duration.

Among SBCS participants who had information on supplement use and comorbidity history available (n = 896), we investigated associations between vitamin D status and regular supplement use in the five years prior to diagnosis (for supplements that may contain vitamin D), and history of diabetes and hypertension, two major comorbidities which have been shown to be associated with vitamin D status in previous reports [25], [26] (**Table 4**). About 17% used calcium supplements regularly, 8.0% used fish oil supplements regularly, 10.8% used multivitamin supplements regularly, 5.8% reported a diagnosis of diabetes, and 20.8% reported a diagnosis of hypertension. Calcium use was suggestively inversely associated with vitamin D deficiency (further-adjusted OR = 0.61, 95% CI: 0.36, 1.05) and multivitamin use was significantly inversely associated with vitamin D deficiency (further-adjusted OR = 0.25, 95% CI: 0.11, 0.58). Diabetes was not associated vitamin D status. Hypertension was associated with both vitamin D insufficiency and deficiency (further-adjusted ORs (95% CIs): 1.91 (1.20, 3.02) and 2.01 (1.16, 3.47), respectively). It should be noted that vitamin D supplement use was uncommon among women residing in Shanghai during the time of this study. Using data from a large prospective cohort study of breast cancer survivors in Shanghai (Shanghai Breast Cancer Survival Study), which includes 780 women from the present study, we found that 0.37% and 0.41% used vitamin D supplements at 6 months and 60 months after diagnosis, respectively. About 21% reported using calcium supplements at 60 months after diagnosis (calcium supplement use was only assessed at the 60-month interview in the Shanghai Breast Cancer Survival Study).

**Table 4:** Prevalence ORs and 95% CIs for the association of supplement use, diabetes and hypertension with vitamin D^a^ status among breast cancer patients (n = 896)^b^.

		Adjusted for age and season						Further adjusted^d^			
Sufficient^c^ N = 552		Insufficient (30–50) N = 938			Deficient (<30) N = 450			Insufficient (30–50) N = 938		Deficient (<30) N = 450	
	NO.	NO.	OR	(95% CI)	NO.	OR	(95% CI)	OR	(95% CI)	OR	(95% CI)
**Calcium supplement use**											
No	199	375	1.00	(reference)	166	1.00	(reference)	1.00	(reference)	1.00	(reference)
Yes	54	75	0.72	(0.48,1.07)	27	0.63	(0.37,1.06)	0.71	(0.47,1.08)	0.61	(0.36,1.05)
**Fish oil use**											
No	234	414	1.00	(reference)	176	1.00	(reference)	1.00	(reference)	1.00	(reference)
Yes	19	36	0.95	(0.52,1.72)	17	1.13	(0.56,2.31)	1.01	(0.55,1.88)	1.18	(0.57,2.45)
**Multivitamin Use**											
No	221	393	1.00	(reference)	185	1.00	(reference)	1.00	(reference)	1.00	(reference)
Yes	32	57	0.90	(0.56,1.46)	8	0.27	(0.12,0.61)	0.87	(0.52,1.44)	0.25	(0.11,0.58)
**Diabetes**											
No	238	424	1.00	(reference)	182	1.00	(reference)	1.00	(reference)	1.00	(reference)
Yes	15	26	0.80	(0.41,1.59)	11	0.87	(0.38,2.02)	0.84	(0.42,1.68)	0.85	(0.36,2.00)
**Hypertension**											
No	218	344	1.00	(reference)	148	1.00	(reference)	1.00	(reference)	1.00	(reference)
Yes	35	106	1.79	(1.15,2.79)	45	1.97	(1.16,3.34)	1.91	(1.20,3.02)	2.01	(1.16,3.47)

a Vitamin D status was based on measured levels in nmol/L.

b This table includes SBCS participants with information collected on supplements, diabetes and hypertension history.

c Reference group.

d Additionally adjusted for education level, total physical activity (MET-hours/day), BMI in quartiles, menopausal status, TNM stage, and tumor estrogen/progesterone receptor status.

## Discussion

In our study of 1940 breast cancer patients, the largest study to date, 23.2% had 25(OH)D concentrations <30 nmol/L (vitamin D deficiency), and 48.4% had 25(OH)D concentrations between 30–50 nmol/L (vitamin D insufficiency). Higher BMI was significantly associated with increased odds of vitamin D deficiency. Total physical activity and biking for transportation were associated with lower odds of vitamin D deficiency. Smoking, although an uncommon behavior in this population (<3% smoked), was associated with both vitamin D insufficiency and vitamin D deficiency. Among participants with available data (n = 896), regular multivitamin supplement use was inversely associated with vitamin D deficiency, and hypertension was associated with increased odds of both vitamin D insufficiency and deficiency.

Specifically for breast cancer patients, while some studies have reported on modifiable lifestyle-related factors in association with circulating 25(OH)D levels in multivariable analyses (i.e., adjusting for potential confounding factors) [8], [14], [16], all studies were limited to Western populations. In our study of breast cancer patients, the first among Chinese women, we found that higher BMI was associated with vitamin D deficiency, as has been found in other studies of breast cancer patients [8], [14], [16]. These findings confirm the well-known associations of lower vitamin D levels with higher BMI observed in healthy adult populations [26]–[28]. Several mechanisms have been proposed to explain the association between obesity and vitamin D, including 1) increased requirement for vitamin D due to deposition of vitamin D in adipose tissue; 2) reduced exposure to sunlight due to lower outdoor physical activity levels; and/or 3) lower levels of 1,25(OH)D among obese individuals [29]. As weight gain after diagnosis of breast cancer is common among breast cancer survivors, our findings of increased odds of vitamin D deficiency among overweight/obese breast cancer patients indicates careful monitoring of vitamin D status and recommendations for supplementation and other lifestyle modifications to improve vitamin D status are warranted in breast cancer survivors.

We identified two studies that investigated multivariable associations between physical activity and vitamin D levels among breast cancer patients. Both included only one measure of physical activity (unclear if recreational or other types of physical activity) and observed that higher levels of physical activity were associated with higher vitamin D levels [8], [16]. In our study, we found that total physical activity and biking for transportation were inversely associated with vitamin D deficiency, adjusting for age, season, BMI, and clinical characteristics. The mechanisms underlying the observed inverse association between physical activity and vitamin D are not established [29]. To date, evidence for a role of physical activity in modulating vitamin D metabolism is inconsistent [29]. Alternatively, physical activity may be an indicator of exposure to sunlight, which is a major source of endogenous vitamin D [29], [30]. The majority of women in Shanghai participate in outdoor exercise, as gyms are expensive and new to Shanghai. Regular exercise or participation in outdoor activity at 30 minutes/day was only non-significantly associated with vitamin D status in our study, suggesting women, and particularly breast cancer patients, may benefit from more outdoor exposure to maintain adequate vitamin D levels. It is not clear why biking for transportation, but not walking for transportation, was associated with a decreased odds of vitamin D deficiency. It is possible that it may be easier to avoid sun exposure while walking (e.g., through the use of umbrellas), however information was not collected on sunlight protection measures.

Vitamin D supplementation is associated with circulating 25(OH)D levels [8], [16]. We did not specifically collect information on vitamin D supplementation in the SBCS, as use of vitamin D supplements was uncommon in Shanghai during the time period of this study. In a subset of breast cancer cases with information on select types of supplement use, we found that regular use of calcium supplements prior to diagnosis (which generally include vitamin D) was suggestively inversely associated with vitamin D deficiency (17% used calcium supplements), and regular use of multivitamin supplements was significantly inversely associated with vitamin D deficiency.

To our knowledge, this is the largest study to date to investigate correlates of vitamin D status, including lifestyle factors, among breast cancer patients, and also the first study among Chinese women. Circulating 25(OH)D concentrations, which reflect both endogenous and exogenous sources of vitamin D, are known to be a valid and stable marker of vitamin D status [31], [32]. Additional strengths of this study include high quality data collection on lifestyle factors and clinical characteristics, including anthropometrics measured in-person according a standard protocol.

This study utilized existing data collected as part of a previous case-control study; therefore, post-diagnosis information was not available for most lifestyle factors with the exception of anthropometric measurements. In addition, vitamin D status was measured before initiation of radiotherapy and chemotherapy for many patients, which precluded our ability to investigate the association between cancer treatment and vitamin D status. Another limitation of our study is that we did not have a measurement of sunlight exposure and were unable to distinguish between indoor and outdoor exercise. However, the latter may not be a major concern in our study as the vast majority of women in Shanghai who exercise participate in outdoor exercise. We did not have information on vitamin D supplement use, and data on other supplement use was only available for 896 SBCS participants. However, adjustment for multivitamin use or calcium supplement use in this subset of participants did not alter associations (data not shown). Finally, vitamin D levels were measured shortly after diagnosis in our study, and this may have contributed to the lower 25(OH)D levels seen in our study as compared to other reports of breast cancer patients [8], [10], as many women may still have been recovering from breast cancer surgery, and spending less time outdoors. However, it should be noted that vitamin D levels have been shown to be lower in the general population in China compared to the other countries, including the U.S. [28].

In conclusion, in this large study of breast cancer patients, we found that vitamin D deficiency and insufficiency were common in Chinese breast cancer patients. Lower physical activity, higher BMI, and smoking were associated with lower vitamin D levels. Vitamin D deficiency is associated with many adverse health outcomes, and may be associated with poorer breast cancer prognosis. Our results provide support for recommendations to increase vitamin D levels among breast cancer survivors, potentially through vitamin D supplement use and healthy lifestyle changes.

## Supporting Information

Table S1 **Associations of lifestyle factors with 25(OH)D levels (nmol/L) among breast cancer patients (n = 1,940).**
(DOCX)

## References

[1] Holick MF (2003) Evolution and function of vitamin D. Recent Results Cancer Res. 164: 3–28. 10.1007/978-3-642-55580-0_1 12899511

[2] Holick MF (2007) Vitamin D deficiency. N Engl J Med 357: 266–281. 17634462 10.1056/NEJMra070553

[3] Pilz S, Tomaschitz A, Obermayer-Pietsch B, Dobnig H, Pieber TR (2009) Epidemiology of vitamin D insufficiency and cancer mortality. Anticancer Res 29: 3699–3704. 19667167

[4] Gupta D, Vashi PG, Trukova K, Lis CG, Lammersfeld CA (2011) Prevalence of serum vitamin D deficiency and insufficiency in cancer: Review of the epidemiological literature. Exp Ther Med 2: 181–193. 22977487 10.3892/etm.2011.205 PMC3440651

[5] Hines SL, Jorn HK, Thompson KM, Larson JM (2010) Breast cancer survivors and vitamin D: a review. Nutrition 26: 255–262. 20004077 10.1016/j.nut.2009.08.020

[6] Laird E, Ward M, McSorley E, Strain JJ, Wallace J (2010) Vitamin D and bone health: potential mechanisms. Nutrients 2: 693–724. 22254049 10.3390/nu2070693 PMC3257679

[7] Levin GP, Robinson-Cohen C, de Boer IH, Houston DK, Lohman K, et al (2012) Genetic variants and associations of 25-hydroxyvitamin D concentrations with major clinical outcomes. JAMA 308: 1898–1905. 23150009 10.1001/jama.2012.17304 PMC3645444

[8] Neuhouser ML, Sorensen B, Hollis BW, Ambs A, Ulrich CM, et al (2008) Vitamin D insufficiency in a multiethnic cohort of breast cancer survivors. Am J Clin Nutr 88: 133–139. 18614733 10.1093/ajcn/88.1.133 PMC2997620

[9] Freedman DM, Looker AC, Chang SC, Graubard BI (2007) Prospective study of serum vitamin D and cancer mortality in the United States. J Natl Cancer Inst 99: 1594–1602. 17971526 10.1093/jnci/djm204

[10] Goodwin PJ, Ennis M, Pritchard KI, Koo J, Hood N (2009) Prognostic effects of 25 hydroxyvitamin D levels in early breast cancer. J Clin Oncol 27: 3757–3763. 19451439 10.1200/JCO.2008.20.0725

[11] Vrieling A, Hein R, Abbas S, Schneeweiss A, Flesch-Janys D, et al (2011) Serum 25 hydroxyvitamin D and postmenopausal breast cancer survival: a prospective patient cohort study. Breast Cancer Res 13: R74. 21791049 10.1186/bcr2920 PMC3236338

[12] Hatse S, Lambrechts D, Verstuyf A, Smeets A, Brouwers B, et al (2012) Vitamin D status at breast cancer diagnosis: correlation with tumor characteristics, disease outcome, and genetic determinants of vitamin D insufficiency. Carcinogenesis 33: 1319–1326. 22623648 10.1093/carcin/bgs187

[13] Park S, Johnson MA (2005) Living in low-latitude regions in the United States does not prevent poor vitamin D status. Nutr Rev 63: 203–209. 16028564 10.1301/nr.2005.jun.203-209

[14] Friedman CF, DeMichele A, Su HI, Feng R, Kapoor S, et al (2012) Vitamin d deficiency in postmenopausal breast cancer survivors. J Womens Health (Larchmt) 21: 456–462. 22385131 10.1089/jwh.2011.3009 PMC3321674

[15] Yao S, Sucheston LE, Millen AE, Johnson CS, Trump DL, et al (2011) Pretreatment serum concentrations of 25-hydroxyvitamin D and breast cancer prognostic characteristics: a case-control and a case-series study. PLoS One 6: e17251. 21386992 10.1371/journal.pone.0017251 PMC3046139

[16] Jacobs ET, Thomson CA, Flatt SW, Newman VA, Rock CL, et al (2013) Correlates of 25 Hydroxyvitamin D and Breast Cancer Stage in the Women’s Healthy Eating and Living Study. Nutr Cancer 65: 188–194. 23441606 10.1080/01635581.2013.756531 PMC4041275

[17] Zheng W, Long J, Gao YT, Li C, Zheng Y, et al (2009) Genome-wide association study identifies a new breast cancer susceptibility locus at 6q25.1. Nat Genet 41: 324–328. 19219042 10.1038/ng.318 PMC2754845

[18] Shu XO, Long J, Lu W, Li C, Chen WY, et al (2012) Novel genetic markers of breast cancer survival identified by a genome-wide association study. Cancer Res 72: 1182–1189. 22232737 10.1158/0008-5472.CAN-11-2561 PMC3294129

[19] Ainsworth BE, Haskell WL, Whitt MC, Irwin ML, Swartz AM, et al (2000) Compendium of physical activities: an update of activity codes and MET intensities. Med Sci Sports Exerc 32: S498–504. 10993420 10.1097/00005768-200009001-00009

[20] Shin A, Matthews CE, Shu XO, Gao YT, Lu W, et al (2009) Joint effects of body size, energy intake, and physical activity on breast cancer risk. Breast Cancer Res Treat 113: 153–161. 18228135 10.1007/s10549-008-9903-x

[21] Wagner D, Hanwell HE, Vieth R (2009) An evaluation of automated methods for measurement of serum 25-hydroxyvitamin D. Clin Biochem. 42: 1549–1556. 10.1016/j.clinbiochem.2009.07.013 19631201

[22] National Institute of Standards and Technology. Material Details. SRM 972–Vitamin D in Human Serum. Gaithersburg, MD: National Institute of Standards and Technology; 2009.

[23] Ross AC, Manson JE, Abrams SA, Aloia JF, Brannon PM, et al (2011) The 2011 report on dietary reference intakes for calcium and vitamin D from the Institute of Medicine: what clinicians need to know. J Clin Endocrinol Metab 96: 53–58. 21118827 10.1210/jc.2010-2704 PMC3046611

[24] Appropriate body-mass index for Asian populations and its implications for policy and intervention strategies. Lancet 363: 157–163. 14726171 10.1016/S0140-6736(03)15268-3

[25] Baz-Hecht M, Goldfine AB (2010) The impact of vitamin D deficiency on diabetes and cardiovascular risk. Curr Opin Endocrinol Diabetes Obes 17: 113–119. 20150805 10.1097/MED.0b013e3283372859

[26] Martins D, Wolf M, Pan D, Zadshir A, Tareen N, et al (2007) Prevalence of cardiovascular risk factors and the serum levels of 25-hydroxyvitamin D in the United States: data from the Third National Health and Nutrition Examination Survey. Arch Intern Med 167: 1159–1165. 17563024 10.1001/archinte.167.11.1159

[27] Forrest KY, Stuhldreher WL (2011) Prevalence and correlates of vitamin D deficiency in US adults. Nutr Res 31: 48–54. 21310306 10.1016/j.nutres.2010.12.001

[28] McCullough ML, Weinstein SJ, Freedman DM, Helzlsouer K, Flanders WD, et al (2010) Correlates of circulating 25-hydroxyvitamin D: Cohort Consortium Vitamin D Pooling Project of Rarer Cancers. Am J Epidemiol 172: 21–35. 20562191 10.1093/aje/kwq113 PMC2892536

[29] Looker AC (2007) Do body fat and exercise modulate vitamin D status? Nutrition Reviews 65: S124–S126. 17867388 10.1301/nr.2007.aug.s124-s126

[30] Holick MF, Chen TC (2008) Vitamin D deficiency: a worldwide problem with health consequences. Am J Clin Nutr 87: 1080S–1086S. 18400738 10.1093/ajcn/87.4.1080S

[31] Ocke MC, Schrijver J, Obermann-de Boer GL, Bloemberg BP, Haenen GR, et al (1995) Stability of blood (pro)vitamins during four years of storage at −20 degrees C: consequences for epidemiologic research. J Clin Epidemiol 48: 1077–1085. 7775995 10.1016/0895-4356(94)00232-f

[32] Antoniucci DM, Black DM, Sellmeyer DE (2005) Serum 25-hydroxyvitamin D is unaffected by multiple freeze-thaw cycles. Clinical Chemistry 51: 258–261. 15613728 10.1373/clinchem.2004.041954

