# Supplementary material for: Correlates of 25-Hydroxyvitamin D among Chinese Breast Cancer Patients
Source: PLoS One. 2014 Jan 21;9(1):e86467. doi: 10.1371/journal.pone.0086467 (PMC3897707; doi:10.1371/journal.pone.0086467)
Supplement: Table S1 — Associations of lifestyle factors with 25(OH)D levels (nmol/L) among breast cancer patients (n = 1,940). (DOCX) [file pone.0086467.s001.docx]

| **Table S1**. Associations of lifestyle factors with 25(OH)D levels (nmol/L) among breast cancer patients (n=1,940) | | |
| --- | --- | --- |
|  | β | P-value |
| **Waist (cm) (ref. ≤ 73 cm)**^a^ |  |  |
| 73.0-<79.0 | 1.779 | 0.094 |
| 79.0-<85.0 | 0.435 | 0.691 |
| ≥85.0 | -1.655 | 0.143 |
| **Waist-hip ratio (ref. ≤ 0.78)** ^a^ |  |  |
| 0.78-<0.82 | 2.376 | 0.032 |
| 0.82-<0.86 | 0.925 | 0.410 |
| ≥0.86 | -0.776 | 0.503 |
| **BMI (kg/m^2^) (ref. ≤ 23)**^a^ |  |  |
| 23-<27.5 | -0.619 | 0.439 |
| ≥27.5 | -3.472 | 0.005 |
| **Total Physical Activity (MET-hours/day) (ref. ≤ 7.65)** ^b^ |  |  |
| 7.65-<10.6 | 1.094 | 0.295 |
| 10.6-<13.5 | 3.191 | 0.003 |
| ≥13.5 | 2.170 | 0.043 |
| **Exercise/sports duration (minutes/day), (ref. = none)**^c^ |  |  |
| <30 | 0.143 | 0.887 |
| ≥30 | 2.199 | 0.140 |
| **Walking for transportation (minutes/day) (ref. ≤ 30)**^d^ |  |  |
| 30-59 | -0.775 | 0.500 |
| 59-90 | 0.0250 | 0.983 |
| ≥90 | -0.537 | 0.662 |
| **Biking for transportation (minutes/day) (ref. = none)**^d^ |  |  |
| <30 | 5.393 | 0.003 |
| 30-59 | 2.583 | 0.026 |
| ≥60 | 3.952 | <0.001 |
| **Ever regularly smoking (ref. = never)**^b^ | -4.908 | 0.032 |
| **Ever regularly drinking (ref. = never)**^b^ | -1.627 | 0.366 |

^a^Adjusted for age, season, education level, SBCS phase, total physical

activity (MET-hours/day), menopausal status, TNM stage, tumor

estrogen/progesterone receptor status.

^b^Adjusted for age, season, education level, SBCS phase, BMI,

menopausal status, TNM stage, tumor estrogen/progesterone receptor

status, and total physical activity (MET-hours/day) (where applicable).

^c^Adjusted for age, season, education level, SBCS phase, BMI,

menopausal status, TNM stage, tumor estrogen/progesterone receptor

status and non-exercise METs.

^d^Adjusted for age, season, education level, SBCS phase, BMI,

menopausal status, TNM stage, tumor estrogen/progesterone receptor

status, exercise duration, walking duration (where applicable), and

biking duration (where applicable).
